# Recent Development of Graphene Based Electrochemical Sensor for Detecting Hematological Malignancies-Associated Biomarkers: A Mini-Review

**DOI:** 10.3389/fchem.2021.735668

**Published:** 2021-08-25

**Authors:** Shougang Wei, Xiuju Chen, Xinyu Zhang, Lei Chen

**Affiliations:** ^1^ Department of Pediatrics, Yidu Central Hospital, Weifang, China; ^2^ Department of Public Health, Yidu Central Hospital, Weifang, China; ^3^ Shandong Freda Pharmaceutical Group Co., Ltd, Linshu, China; ^4^ Key Laboratory of Biopharmaceuticals, Engineering Laboratory of Polysaccharide Drugs, Shandong Academy of Pharmaceutical Sciences, Jinan, China

**Keywords:** hematological malignancies, leukemia, graphene, electrochemical sesnor, DNA biosensors, label-free biosensors

## Abstract

Hematologic malignancies are a group of malignant diseases of the hematologic system that seriously endanger human health, mainly involving bone marrow, blood and lymphatic tissues. However, among the available treatments for malignant hematologic diseases, low detection rates and high recurrence rates are major problems in the treatment process. The quantitative detection of hematologic malignancies-related biomarkers is the key to refine the pathological typing of the disease to implement targeted therapy and thus improve the prognosis. In recent years, bioelectrochemical methods for tumor cell and blood detection have attracted the attention of an increasing number of scientists. The development of biosensor technology, nanotechnology, probe technology, and lab-on-a-chip technology has greatly facilitated the development of bioelectrochemical studies of cells, especially for blood and cell-based assays and drug resistance differentiation. To improve the sensitivity of detection, graphene is often used in the design of electrochemical sensors. This mini-review provides an overview of the types of hematological malignancies-associated biomarkers and their detection based on graphene assisted electrochemical sensors.

## Introduction

Hematological malignancies are a series of blood disorders characterized by the malignant transformation of normal cells of bone marrow and extramedullary hematopoietic organs into a large number of tumor cells, which can seriously threaten human health (Chen et al., 2019; Gupta et al., 2020). They are heterogeneous diseases, mainly classified as Leukemia, Lymphoma and Multiple myeloma, and include more than 30 different subtypes. These subtypes differ greatly in terms of etiology, clinical manifestations, diagnosis and treatment, and prognosis. Therefore, a refined stratified diagnosis of the disease is needed to implement targeted therapy and thus improve the prognosis (Fracchiolla et al., 2013; Hianik, 2021; Luo et al., 2021; Zhang et al., 2021). Currently, the clinical diagnosis of hematologic tumors relies on imaging, including X-ray, computed tomography, magnetic resonance imaging, endoscopy, and ultrasound. Although clinical imaging techniques can “locate and diagnose” tumors, they have low sensitivity and limited ability to differentiate between benign and malignant lesions (Baldo et al., 2016; Hasanzadeh et al., 2017; Karimi-Maleh et al., 2021). However, these methods have certain limitations: for example, high cost, low sensitivity, long detection period, expensive, complicated detection methods, and some detection methods even have radioactive contamination. Compared with these methods, bioelectrochemical methods have the advantages of simplicity, high sensitivity and low cost. It can be easily implemented for quantitative determination and clinical application of blood samples (Miao et al., 2019; Rasheed et al., 2020; Li et al., 2021; Liu et al., 2021).

With the rapid development of modern biomedical technologies such as molecular biology and genomics, people have gained a deeper understanding of the pathogenesis of hematologic tumors and have discovered a variety of tumor biomarkers related to hematologic tumors. Information about the currently used hematologic tumor-related biomarkers is shown in Table 1.

**TABLE 1:** Currently used hematologic tumor-related biomarkers.

Hematological malignancies	Subtype	DNA	miRNA	Protein	Cell
Lymphoma	NHL	C-myc, Bcl-, Bcl-6, Bcl-11, P53, P16, B2M, PTPN1, TNFAIP3	miR-21, miR-155	C-myc, Bax, Bcl2, CD3/4/7/8/20/22/45	Ramos
	HL	B2M, PTPN11, TNFAIP3	-	CD30/68/80	R-S
Leukemia	AML	PML/RARα, AML1-ETO, CBFβ-MYH11	miR-181a, miR-15	CD33	HL-60/K562
	ALL	FLT3, PRAME, NPM1, C-KIT, CEBPA	miR-128/181/204/218/221/331	CD10, CD19	CCRF-CEM
	CML	P185BCR/ABL, TEL/AML1, E2A/PBX1, MLL/AF4	miR-217/221/222 miR-17-92	ABL	K562
	CLL	PRAME/BCR/ABL, ASS	miR-15/16/29/181	CD52	CLL
Multiple myeloma	-	KRAS, NRA, BRAF, TRAF3, CYLD, LTB, TP53, ATM, ATR	miR-21/32/93/133 miR-206/221/222, miR-17-92	CD30/38, M protein, CRP, LDH	MM

The introduction of nanomaterials in electrochemical analysis increases the conductivity and surface area of electrodes, thus improves the catalytic activity. These properties can improve the sensor detection sensitivity, shorten the response time, and realize the real-time monitoring of the detector (Gurudatt et al., 2019; Hrichi et al., 2019). Nanomaterials are modified onto the surface of electrochemical sensors to capture biomolecules and improve the immobilization efficiency and the sensitivity of the sensors. In addition, nanomaterials can be used as markers to label and can maintain the biological activity of biomolecules and their corresponding components.

Graphene, with its unique physical and chemical properties, is widely used in electrochemical analysis and has become a research hotspot for electrochemical sensors. For example, Ji et al. (2014) used graphene as a sensor interface to capture more antibodies and thus amplify the detection signal. The good performance of the sensor is mainly attributed to the high specific surface area of graphene. The strong adsorption property can immobilize more biomolecules. Its good electrical conductivity improves the electron transfer. Hong et al. (2015); Zhang (2016); Xu et al. (2017) constructed a series of electrochemical immunosensors based on sulfur corynes/graphene complexes as immobilization platforms for primary antibodies. All of these sensors showed a wide detection range, low detection limits and good stability. In addition, reduced graphene can also be applied for the preparation of electrochemical sensors (Yang and Zhang, 2014). Haque et al. Ensafi et al. (2016) prepared an N-acryloxysuccinimide activated amphiphilic polymer-coated reduced graphite oxide as a sensing interface for electrochemical immunosensors. In this paper, we discuss the application of bioelectrochemistry in the detection of hematological malignancies from different perspectives of graphene assisted electrochemical and electrogenerated chemiluminescence biosensors.

## Labeled Biosensors

A single target is selected for sensing detection, which is a single-target analysis method. Its direct quantification produces a signal with low interference, good selectivity, high sensitivity and fast response, which can detect the target at trace level. Labeled biosensors are used for quantitative detection by monitoring the change in signal when a marker interacts with a target. The commonly used markers include quantum dots, metal ions, ferrocene, methylene blue, sulfur cordial, anthraquinone and enzymes with redox activity.

DNA electrochemical sensors are used to detect targets by hybridization reactions of nucleic acid DNA. Usually, a single-stranded DNA (ssDNA) probe is immobilized on the electrode surface, and the complementary sequence-specific hybridization between the probe and the target further captures the target DNA (tDNA) into a double-stranded structure (dsDNA), which triggers a change in the electrochemical signal for quantitative analysis of tDNA.

Wang X. et al. (2018) proposed a novel and sensitive electro-generated chemiluminescence (ECL) biosensing system for the detection of the p16INK4a gene. A nanofiber composite was synthesized using graphene as the backbone of pyrrole. This composite serves as a carrier for labeling dsDNA. after optimization, this ECL can detect p16INK4a linearly in the range of 0.1 pM–1 nM, and the detection limit can reach 0.05 pM. Mazloum-Ardakani et al. (2018) reported a biosensor with excellent electrical properties for the detection of acute lymphoblastic leukemia. This sensor does not require the involvement of enzymes and only requires the use of a simple one-step synthesis of poly(catechol). By modification with graphene sheets and AuNPs, the electrical conductivity of poly(catechol) can be greatly enhanced. In this sensor, catechols are used as active probes. Under optimal conditions, the logarithm of this sensor is linear for the target DNA concentration in the range of 100.0 μM to 10.0 pM.

Graphene was also used to prepare electrochemical sensors to detect BCR/ABL fusion genes (Wang et al., 2014). Chitosan (CS) was used to prepare a graphene sheet suspension and modified on GCE, electropolymerized to form a PANI layer and then solidly loaded with AuNPs for the capture probe. The probes were double-labeled with 5′-SH and 3′-biotin for hairpin structure. After hybridization with the target DNA, the hairpin structure is forced open and the streptavidin-alkaline phosphatase can covalently bind to the probe via avidin. Then, the catalytically electroactive 1-naphthyl phosphate can be hydrolyzed to 1-naphthol and exhibit a reduction current for detection. Under optimal conditions, the sensor allows linear detection of the BCR/ABL fusion gene in the range of 10–1000 pM with a detection limit of 2.11 pM.

Several miRNAs are also important markers for the detection of hematological malignancies. Nowadays electrochemical techniques often use sandwich method via nucleotide hybridization to achieve miRNA detection. Cheng et al. (2015) prepared AuNPs-graphene modified GCE, assembled DNA1 to capture the target miRNA-21, and further bound DNA2 to form a sandwich structure. They then used Cd^2+^-modified titanium phosphate nanoparticles as signal probes, along with Ru(NH_3_)_6_^3+^ as the electron mediator, bound electrostatically to the sandwich structure. This sensor has a detection linear range of 10^−18^ to 10^−11^ M with a low limit of detection of 0.76 aM. The performance of some other sensors composed of graphene for miRNA detection was listed in Table 2.

**TABLE 2:** Electrochemical sensor for RNA biomarkers in hematological malignancies.

Target	Sensor	Linear range	LOD	References
miRNA-21	Au-RGO/TiPCd^2+^/Ru(NH_3_)_6_^3+^	1 aM–10 pM	0.76 aM	Cheng et al. (2015)
miRNA-21	Graphene/GME	-	3.12 pM	Kilic et al. (2015)
miR-155	SS-probe/GO/GNR	2.0 fM–8.0 pM	0.6 fM	Azimzadeh et al. (2016)
miR-155	Amino-graphene	30 pM–1 nM	12.5 pM	Salimi et al. (2019)
miR-122	Graphene-PGE	0.5–7 μg/ml	1.06 pM	Kilic et al. (2016)

Protein-based markers are based on the principle of specific binding between antigen and antibody to form stable antigen-antibody complexes, which are converted into detectable electrochemical signals for qualitative and quantitative analysis and detection. He et al. (2013) developed a graphene-assisted electrochemical immunoassay platform for the detection of c-Myc. AuNPs were used as a label for the determination of c-Myc. This sensor is based on a sandwich immunoassay strategy where the target c-Myc is captured by an antibody to c-Myc modified on a gold substrate, followed by the addition of another CAb conjugated to the AuNPs label. sensor allows linear detection of c-Myc between 4.3 Pm–43 nM with a detection limit of 1.5 pM.

Cellular biosensors often employ antigenic antibodies as recognition elements for cellular detection. The target is captured by a specific immune reaction with certain groups on the cell surface and converted into a detectable electrochemical signal for qualitative and quantitative analysis. Yang et al. (2013) assembled a cell sensor using carboxymethyl chitosan-functionalized graphene (CMC-G). This sensor exhibited good electrochemical behavior and cell capture ability for HL-60 cells. Wang J. et al. (2018) proposed an electrochemical biosensor assembled by signal amplification strategy using graphene oxide - polyaniline (PANI) as a modified material for the detection of K562 cells. Polystyrene microspheres functionalized with multilayer CdS QDs were used as a biosensor. the modification of GO-PANI composite not only improved the electron transfer rate but also increased the loading rate of tumor cells. This electrochemical sensor can detect a minimum of three K562 cells.

Recently, aptamers are also often used as recognition elements for cellular assays. Aptamers are short, single-stranded oligonucleotides that are usually highly affinity and specific for the target. Aptamers are smaller and more stable than traditional biomolecular ligands such as antibodies, allowing for better selective recognition of target tumor cells. Liu et al. (2016) found that the morphology of AuNPs can affect the catalytic activity of graphene towards peroxidase mimics. They synthesized Au flowers *in situ* on the hemin/RGO surface and solidified the aptamer. Then, they used this composite to prepare an electrochemical sensor for K562 leukemia cancer cells. Zheng et al. (2019) developed an electrochemical sensor for the highly sensitive detection of K562 in tumor cells. They first designed aptamer-DNA concatamer-CdTe QDs probes by DNA hybridization and covalent assembly. Then, they assembled GCE/GO/PANI/GA/Concanavalin A step by step. this electrochemical sensor can reach the detection limit of 60 cells/ml.

## Label-free Electrochemical Sensors

Label-free electrochemical sensing methods are mostly used for the detection of DNA and CTC markers in blood tumors. These sensors are generally based on chemical methods to bind the target by immobilizing the capture probe on the electrode surface through covalent linkage, including Au-S bonding, amino-carboxy and amino-sulfonic acid groups. In addition, binding is also based on physical methods such as electrostatic adsorption and amino acid functionalized cross-linking.

**FIGURE 1:**
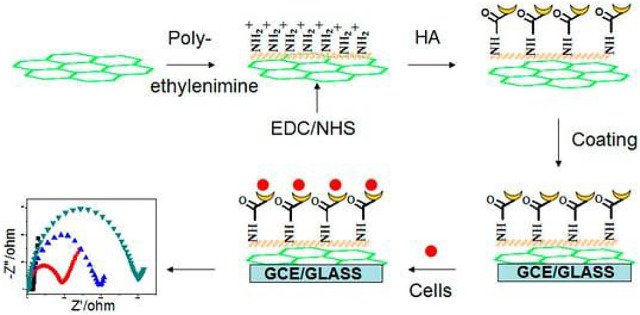
Scheme of hyaluronate-functionalized graphene for label-free HCT-116 detection (Copyright obtained from MDPI.)

Jing et al. (2018) prepared an effective hyaluronate-functionalized graphene (HG) by chemical reduction of GO. Using the self-assembly of HG with ethylenediamine and sodium hyaluronate, a label-free electrochemical impedance spectroscopy cell sensor could be assembled. Under optimal conditions, this sensor can provide highly sensitive detection of cancer cells HCT-116. Figure 1 shows a schematic diagram of this detection strategy.

Shamsipur et al. (2020) exfoliated graphene with gold nanoclusters (Hb@AuNCs) capped with hemoglobin. This graphene has good dispersion due to the fact that Hb@AuNCs can act as stabilizers through non-covalent bonds. This graphene was used to make a highly sensitive electrochemical sensor. Highly sensitive detection of BCR/ABL fusion gene can be performed based on “signal off” and “signal on” strategies”. Under optimal conditions, the sensor can detect linearly in the range of 0.1 fM to 10 pM. The detection limit is 0.030 fM.

Zhang et al. (2013) proposed a label-free electrochemical sensor consisting of a combination of GO and polylysine that can be used for the detection of K562 cells. This sensor is a thin film that can immobilize live cells very well. [Fe(CN)_6_]^3-/4^- was used as a detection probe and EIS was used as a technique for the detection. Under optimal conditions, the electron transfer resistance correlates well with the logarithmic value of the K562 cell concentration. The detection range of this sensor was from 100-10^7^ cell/mL. The detection limit was 30 cell/mL.

Label-free electrochemical sensors save time and cost by detecting the target material directly without a marker. This can maintain the affinity of the recognition element to the target and thus avoid the interference caused by secondary detection and labeling reagents. However, label-free electrochemical sensors have some drawbacks in practice, such as the possibility of detachment of the recognition element bound to the electrode surface during the experiment and the fluctuation of the EIS, resulting in errors. These problems can lead to relatively poor accuracy in the actual test and limit its practical application.

## Conclusion

This mini-review presents an introduction to biomarkers and electrochemical detection techniques in hematological malignancies. Among them we focus on the impact of graphene development in the construction of such electrochemical sensors. However, improving the sensitivity, specificity, and practicality of graphene electrochemical biosensing methods in the face of biomarkers present at trace levels in actual clinical samples is still an important current issue. It is clear from the content that the current graphene electrochemical sensor can detect relatively few markers, so more markers can be developed for detection. Graphene-based electrochemical sensors in hematological malignancies biomarker analysis is still focus on the single marker detection. Therefore, selecting multiple types of typical markers for specific tumors for combined detection can make the assay more specific and improve the sensitivity and detection rate of tumor detection. Meanwhile, current electrochemical biosensors are not ideal for the detection of marker molecules in population samples. Effective coupling of sensing design with population sample pre-treatment, integrating target extraction, enrichment and detection, and effectively improving the utility of the sensor will be the focus of future research.
